# Hypertension and risk of psoriasis incidence: An 11-year nationwide population-based cohort study

**DOI:** 10.1371/journal.pone.0202854

**Published:** 2018-08-24

**Authors:** Ha-Na Kim, Kyungdo Han, Sang-Wook Song, Ji Hyun Lee

**Affiliations:** 1 Department of Family Medicine, St. Vincent’s Hospital, College of Medicine, The Catholic University of Korea, Seoul, Republic of Korea; 2 Department of Biostatistics, College of Medicine, The Catholic University of Korea, Seoul, Republic of Korea; 3 Department of Dermatology, Seoul St. Mary’s Hospital, College of Medicine, The Catholic University of Korea, Seoul, Republic of Korea; University of Naples, ITALY

## Abstract

Psoriasis is a chronic inflammatory skin disease that is characterized by T-cell mediated immune response, and has been known to increase the risk of developing hypertension. However, the risk of psoriasis in patients with hypertension is not clear. Therefore, we investigated the risk of psoriasis in patients with hypertension. A total of 256,356 adults (42,726 in the hypertension group and 213,630 in the control group) were followed from 2003 to 2013 in a nationwide population-based cohort study. During the follow-up, 9,254 participants (3.6%) were found to have psoriasis (2,152 [5.0%] in the hypertension group and 7,102 [3.3%] in the control group). The hypertension group had a higher risk of psoriasis incidence (hazard ratio [HR] 1.54, 95% confidence interval [CI] 1.47–1.61, *P* < 0.001), and the association remained significant after adjusting for comorbidities of diabetes and dyslipidemia, antihypertensive medication and nonsteroidal anti-inflammatory drug use, and sociodemographic factors (HR 1.18, 95% CI 1.08–1.28, *P* < 0.001). In conclusion, hypertension was significantly associated with an increased risk of psoriasis incidence. Further studies are needed to confirm whether hypertension is associated with the incidence of psoriasis.

## Introduction

Psoriasis is a chronic inflammatory skin disease related T-cell mediated immune mechanisms [1]. The prevalence of psoriasis is approximately 2% of the world’s population [1], and is relatively lower in Asians, including Korea [2–5]. However, psoriasis has major effects on life since it is not only results in functional and social morbidities with socioeconomic cost [6], but also associated with an increased risk of various accompanying diseases [7,8]; thus, psoriasis has become a medical concern.

Hypertension, a well-known cardiovascular risk factor, contributes to the development of myocardial ischemia and infarction, stroke, and cardiovascular death [9]. Several studies have reported positive associations between psoriasis and hypertension [10,11]. Furthermore, most studies show that patients with psoriasis have a higher risk of hypertension [10,12]. However, prospective studies investigating the association between hypertension and the risk of psoriasis development are rare. Wu et al. conducted a prospective study of the association between hypertension and the risk of psoriasis in US women [13], but no studies to date have evaluated the risk of psoriasis in men as well as women with hypertension.

Therefore, we investigated the association between hypertension and the prospective risk of psoriasis development using the National Health Insurance Service National Sample Cohort (NHIS-NSC) 2002–2013 that was produced by the Korean National Health Insurance Service (KNHIS).

## Materials and methods

### Ethical approval

The data used were extracted from the KNHIS claim database. All data were anonymized and provide us except for the patient’s personal information. This study was approved by the Institutional Review Board of the KNHIS (NHIS-2016-2-213). In addition, the Ethics Committee of Seoul St. Mary’s Hospital, the Catholic University of Korea approved this study design and a waiver of informed consent for the study (KC16EISE0524).

### Data sources

In Korea, all of the nationals are obligated to enroll in the KNHIS, a nationwide health insurance system. Thus, the health system data are centralized in large databases. Medical providers are required to submit claims regarding diagnostic codes, procedures, prescription, the patient’s personal information, and medical costs. The health care records of patients are not duplicated or omitted because all Korean nationals receive a unique identification number at birth, and the KNHIS uses the standard codes of the International Statistical Classification of Diseases and Related Health Conditions, 10th revision (ICD-10). This study used data from the NHIS-NSC 2002–2013, which were produced by the KNHIS using a proportionate stratified random sampling method based on 1476 strata according to age, sex, and household income to generate a nationally representative sample from a total of 46,605,433 Korean individuals in 2002. Therefore, the data comprised 1,025,340 subjects, which is approximately 2.2% of the entire Korean population.

The KNHIS converted all personal identification numbers into surrogate numbers before releasing the data file to researchers as a scrambled secondary data file to secure the privacy of insured persons.

### Study population

The hypertension group included all patients initially diagnosed with hypertension (ICD-10: I10–I15) between January 2003 and December 2005 (n = 110,393). We excluded those patients aged < 20 years, those who were diagnosed with hypertension and/or psoriasis prior to enrollment, and those who had been diagnosed with psoriasis prior to their hypertension diagnosis and had a one-year washout period from January to December 2002 to reduce confounding of previously diagnosed hypertension. Finally, the hypertension group consisted of 42,726 subjects (23,383, 10,732, and 8,611 subjects in 2003, 2004, and 2005, respectively) who were considered new incident cases of hypertension. For the control group, 213,630 subjects (five per patient with hypertension) were selected from the database; they were matched to the patients with hypertension according to age and sex using propensity score matching. All of the included subjects were tracked on the basis of their health care records during the 11-year period from 2003–2013 to identify patients who developed psoriasis (ICD-10: L40).

### Covariates

Information on age (20–39, 40–64, and ≥ 65 years), sex, residential area (urban area including Seoul city or rural area), household income (≤ 30% and > 30% of the median), use of antihypertensive medications including angiotensin-converting enzyme inhibitors, angiotensin receptor blockers (ARBs), ß-blockers, calcium channel blockers (CCBs), and thiazides, and use of nonsteroidal anti-inflammatory drugs (NSAIDs) was obtained from the baseline. History of comorbidities including dyslipidemia and diabetes mellitus were defined as a diagnosis of these conditions between 2003 and 2013 prior to the diagnosis of psoriasis.

### Statistical analysis

The characteristics of the study population according to hypertension status were analysed using the chi-squared test. Univariate and multivariate Cox proportional hazard regression analyses were conducted to identify the association between hypertension and the prospective development of psoriasis. A multivariate Cox proportional hazard regression analysis was used to adjust for comorbidities, antihypertensive medication and NSAID use, age, sex, residential area, and household income, which were obtained from baseline. Psoriasis-free survival was analysed using the Kaplan-Meier method for the 11-year follow-up period, and log-rank tests were performed to analyse the differences in psoriasis incidence between the hypertension and comparison groups. All of the statistical analyses were performed using the SAS software (version 9.4, SAS Institute, Cary, NC, USA). *P* values less than 0.05 were considered statistically significant.

## Results

### Characteristics of the study population

During the follow-up period, 9,254 psoriasis cases occurred among 256,356 subjects. Table 1 shows the characteristics of the study population for the hypertension and comparison groups. The incidence of psoriasis in the hypertension group was higher than that in the comparison group (5.0% vs. 3.3%, *P* < 0.001). No differences in age, sex, or residential area were detected between the subjects with and without hypertension, but there were significant differences in the prevalence of diabetes and dyslipidemia, antihypertensive medication and NSAID use, and household income between the two groups.

**Table 1:** Characteristics of the study population.

	Hypertension	Comparison	
	(n = 42,726)	(n = 213,630)	*P*
New-onset psoriasis	2,152 (5.0)	7,102 (3.3)	< 0.001
Year of hypertension diagnosis			-
2003	23,383 (54.7)	116,915 (54.7)	
2004	10,732 (25.1)	53,660 (25.1)	
2005	8,611 (20.2)	43,055 (20.2)	
Age (year)			0.798
20–39	4,317 (10.1)	21,585 (10.1)	
40–64	29,303 (68.6)	146,208 (68.4)	
≥ 65	9,106 (21.3)	45,837 (21.5)	
Male	21,912 (51.3)	109,867 (51.4)	0.587
Residential area (urban)	19,566 (45.8)	97,460 (45.6)	0.512
Low income^a^	6,618 (15.5)	43,488 (20.4)	< 0.001
Medication use			
ACEi	10,683 (25.0)	223 (0.1)	< 0.001
ARB	10,525 (24.6)	80 (0.04)	< 0.001
ß-blocker	15,824 (37.0)	3,031 (1.4)	< 0.001
CCB	22,111 (51.8)	2,328 (1.1)	< 0.001
Thiazide	17,981 (42.1)	6,412 (3.0)	< 0.001
NSAID	29,946 (70.1)	110,986 (52.0)	< 0.001
Comorbidities			
Diabetes	5,933 (13.9)	7,172 (3.5)	< 0.001
Dyslipidemia	5,945 (13.9)	4,367 (2.2)	< 0.001

Values are expressed as n (%).

^a^Low income is defined as household income ≤ 30% of the median.

Abbreviations: ACEi, angiotensin-converting enzyme inhibitor; ARB, angiotensin receptor blocker; CCB, calcium channel blocker; NSAID, nonsteroidal anti-inflammatory drug.

### Hypertension and other factors as predictors of psoriasis incidence

As shown in Table 2, the group with hypertension had a higher risk of developing psoriasis (hazard ratio [HR] 1.54, 95% confidence interval [CI] 1.47–1.61, *P* < 0.001), and this association remained significant after adjusting for age, sex, household income, residential area, comorbidities of diabetes and dyslipidemia, NSAID use, and antihypertensive medication (HR 1.18, 95% CI 1.08–1.28, *P* < 0.001). The use of CCBs or thiazides was associated with a higher risk of psoriasis incidence after adjusting for the aforementioned covariates (HR 1.14, 95% CI 1.05–1.23, *P* = 0.002, and HR 1.10, 95% CI 1.02–1.18, *P* = 0.010, respectively).

**Table 2:** Longitudinal association between hypertension and psoriasis incidence.

	Univariate Cox			Multivariate Cox^a^		
	HR	95% Cl	*P*	HR	95% Cl	*P*
Hypertension	1.54	1.47–1.61	< 0.001	1.18	1.08–1.28	< 0.001
Age (year)						
20–39	1			1		
40–64	1.35	1.24–1.45	< 0.001	1.32	1.22–1.43	< 0.001
≥ 65	1.26	1.16–1.38	< 0.001	1.27	1.16–1.39	< 0.001
Male	1.10	1.05–1.14	< 0.001	1.17	1.12–1.22	< 0.001
Antihypertensive medication						
ACEi	1.46	1.34–1.59	< 0.001	1.07	0.99–1.16	0.102
ARB	1.55	1.43–1.69	< 0.001	1.00	0.91–1.10	0.979
ß-blocker	1.45	1.36–1.55	< 0.001	1.08	0.98–1.20	0.115
CCB	1.55	1.46–1.64	< 0.001	1.14	1.05–1.23	0.002
Thiazide	1.48	1.40–1.57	< 0.001	1.10	1.02–1.18	0.010
Diabetes	1.34	1.24–1.46	< 0.001	1.08	0.99–1.17	0.095
Dyslipidemia	1.64	1.51–1.78	< 0.001	1.28	1.17–1.40	< 0.001

^a^Multivariate Cox regression analyses are adjusted for age, sex, household income, residential area, comorbidities of diabetes and dyslipidemia, current nonsteroidal anti-inflammatory drug use, and antihypertensive medication.

Abbreviations: ACEi, angiotensin-converting enzyme inhibitor; ARB, angiotensin receptor blocker; CCB, calcium channel blocker; CI, confidence interval; HR, hazard ratio.

### Cumulative impact of hypertension on psoriasis incidence

**Fig 1:**
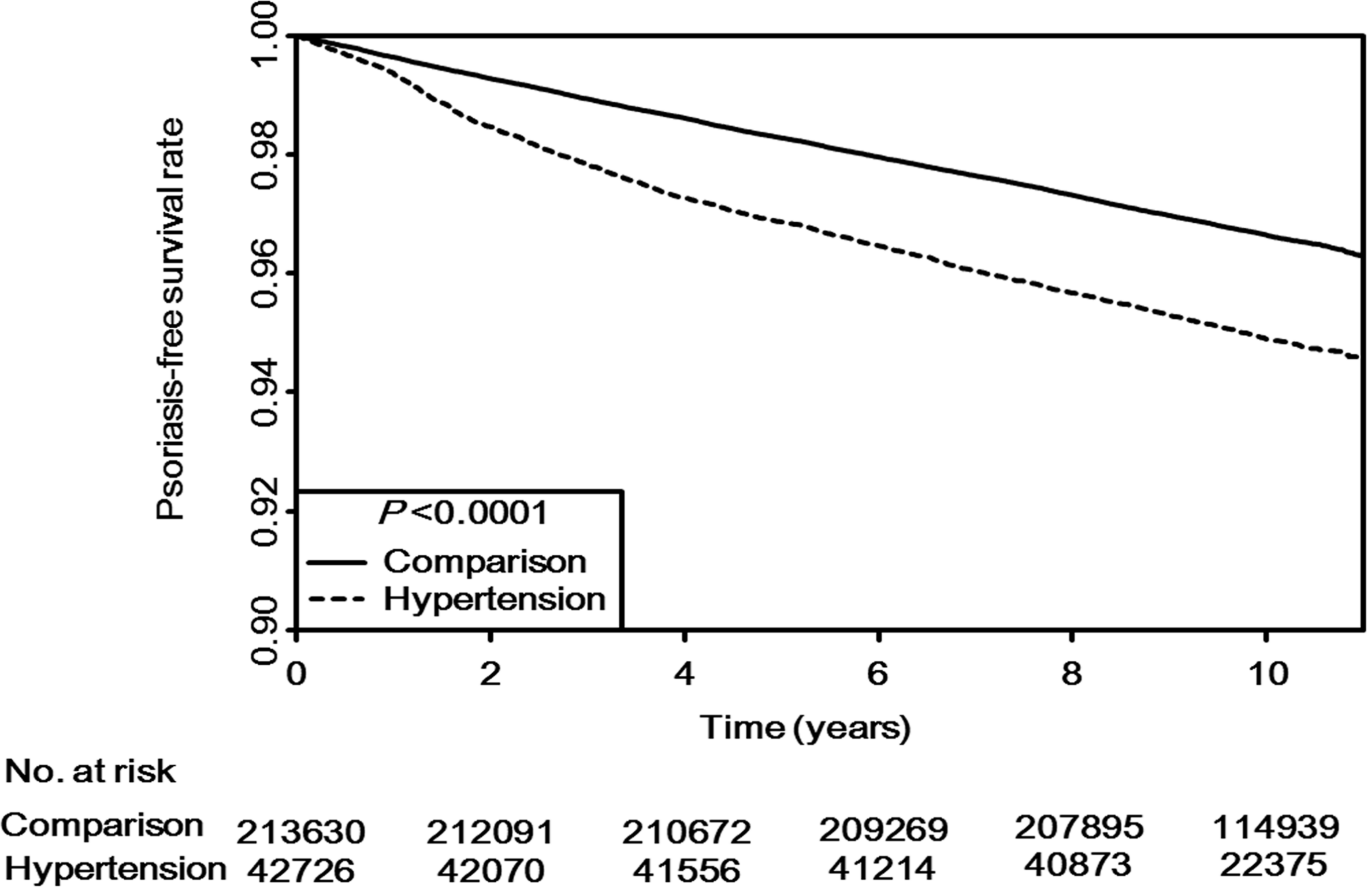
Kaplan–Meier survival curve. The overall psoriasis-free survival analyses for the hypertension and comparison groups are shown. The log-rank *P* value is significant, indicating a significant difference in psoriasis incidence between the hypertension and comparison groups.

The results of Kaplan-Meier curves for the psoriasis-free survival rate are shown in Fig 1. The median follow-up duration was 9.7 years and 2,487,617 person-years were examined (409,565 person-years for the hypertension group and 2,078,052 person-years for the comparison group). Psoriasis occurred at rates of 10.5 and 6.8 per 1000 person-years in the hypertension and comparison groups, respectively. Psoriasis occurred more frequently in the hypertension group than in the comparison group (*P* < 0.001 by log-rank test).

### Subgroup analysis of the effects of hypertension on psoriasis incidence

The subgroup analyses of age and sex are shown in Table 3. Both males and females with hypertension had increased development of psoriasis after multivariate adjustments. The subjects with hypertension aged < 65 years had a higher risk of psoriasis development. Both male and female subjects with hypertension aged < 65 years had a higher risk of developing psoriasis, but males and females aged ≥ 65 years did not. ARB use was associated with a higher risk of psoriasis incidence among males aged < 65 years, and ß-blocker use was associated with an increased risk of psoriasis among subjects aged ≥ 65 years. Associations were found between the use of CCBs or thiazides and the risk of psoriasis among females aged < 65 years.

**Table 3:** Longitudinal association between hypertension and psoriasis incidence according to age and sex.

	Sex		Age		Age < 65 years		Age ≥ 65 years	
	Male	Female	< 65 years	≥ 65 years	Male	Female	Male	Female
Hypertension	1.14(1.01–1.29)	1.19(1.05–1.35)	1.20(1.09–1.32)	1.06(0.89–1.27)	1.16(1.01–1.33)	1.23(1.07–1.42)	1.06(0.82–1.37)	1.07(0.83–1.36)
Antihypertensive medication								
ACEi	1.09(0.98–1.25)	0.92(0.79–1.06)	0.99(0.89–1.11)	1.04(0.85–1.27)	1.09(0f.93-1.26)	0.90(0.76–1.07)	1.12(0.84–1.50)	0.98(0.74–1.30)
ARB	1.19(1.04–1.36)	0.99(0.85–1.14)	1.05(0.94–1.18)	1.14(0.90–1.43)	1.17(1.01–1.37)	0.95(0.81–1.12)	1.22(0.87–1.70)	1.07(0.78–1.48)
ß-blocker	1.09(0.97–1.22)	1.05(0.94–1.18)	1.03(0.94–1.13)	1.21(1.02–1.42)	1.06(0.93–1.20)	1.01(0.89–1.14)	1.22(0.96–1.56)	1.20(0.96–1.51)
CCB	1.11(0.99–1.24)	1.19(1.07–1.33)	1.20(1.09–1.31)	0.98(0.83–1.15)	1.18(1.01–1.30)	1.27(1.11–1.45)	0.98(0.77–1.23)	0.98(0.79–1.23)
Thiazide	0.98(0.87–1.09)	1.22(1.11–1.34)	1.14(1.05–1.24)	1.00(0.87–1.15)	0.99(0.88–1.13)	1.28(1.14–1.43)	0.92(0.74–1.14)	1.07(0.88–1.28)
Diabetes	1.07(0.95–1.19)	1.13(0.99–1.29)	1.07(0.96–1.18)	1.15(0.98–1.35)	1.03(0.90–1.18)	1.12(0.96–1.31)	1.16(0.93–1.45)	1.14(0.98–1.43)
Dyslipidemia	1.26(1.11–1.43)	1.31(1.16–1.49)	1.28(1.16–1.41)	1.34(1.09–1.64)	1.33(1.16–1.52)	1.21(1.05–1.40)	0.91(0.64–1.31)	1.68(1.31–2.15)

Values are expressed as hazard ratios (95% confidence interval). Cox regression analyses are adjusted for age, sex, household income, residential area, comorbidities of diabetes and dyslipidemia, nonsteroidal anti-inflammatory drug use, and antihypertensive medication.

Abbreviations: ACEi, angiotensin-converting enzyme inhibitor; ARB, angiotensin receptor blocker; CCB, calcium channel blocker.

## Discussion

In this first large-scale cohort study, which included 42,726 subjects with hypertension and 213,630 matched controls, hypertension was positively associated with an increased risk of psoriasis development in the 11-year follow-up period, after adjusting for comorbidities of diabetes or dyslipidemia, antihypertensive medication and NSAID use, and sociodemographic factors. In subgroup analysis according to sex and age, the causal association between hypertension and risk of psoriasis was similar, but hypertension in subjects aged ≥ 65 years was not associated with psoriasis incidence. CCB and thiazide use was associated with an increased risk of psoriasis incidence.

It has been widely reported that psoriasis is associated with metabolic components including diabetes, hypertension, and obesity. Psoriasis and these components may share similar risk factors such as chronic inflammation and oxidative stress, deviant angiogenesis, and specific genetic factors [14]. A number of previous studies have also reported that hypertension is positively associated with psoriasis [10,15–18]. Indeed, several biological pathways have been implicated in the association between psoriasis and hypertension including overexpression of endothelin in vascular endothelial cells and keratinocytes, increased oxidative stress, and common inflammatory mechanisms such as tumor necrosis factor and interleukin (IL)-17 [19–23]; however, previous prospective cohort studies investigating the association between hypertension and the risk of psoriasis development are rare [13]. Several studies have proposed a potential mechanism underlying the positive association between hypertension and the risk of psoriasis incidence. Cheng et al. showed that the *leucyl/cystinyl aminopeptidase (LNPEP)* gene was significantly downregulated in psoriasis lesions, and identified a coding variant of *LNPEP* in Chinese individuals with psoriasis, supporting the involvement of *LNPEP* in the etiology of psoriasis [24]. LNPEP (also known as angiotensin IV receptor), vasopressinase, and insulin-regulated aminopeptidase are aminopeptidases encoded by the *LNPEP* gene and are important components of the renin–angiotensin system pathway. A genetic variation in *LNPEP* was associated with plasma vasopressin clearance and serum sodium regulation [25], which play similar roles in the pathogenesis of hypertension and psoriasis. Furthermore, angiotensin IV, via angiotensin IV receptors, reportedly activates the nuclear factor kappa-light-chain-enhancer of activated B cells (NF-κB) pathway and increases expression of pro-inflammatory genes [26], similar to the pathogenesis of psoriasis, which involves NF-κB and IL-23 signaling [27]. Elevated plasma renin and/or angiotensin-converting enzyme activities, which are associated with hypertension, were observed in psoriasis patients [28]. In addition, angiotensin II not only leads to the activation of physiological processes that contribute to increased blood pressure, but also stimulates T cell proliferation and promotes inflammation [29]; therefore, it may play an important role in the development of inflammatory and immune-mediated skin lesions in psoriasis.

The use of antihypertensive medications may be linked to increased incidence or exacerbation of psoriasis [30,31], but prospective studies concerning the causal relationship between antihypertensive medication use and psoriasis incidence are scarce. A previous prospective study in 77,728 American women showed that long-term hypertensive status and use of ß-blockers for 6 years or more were associated with a risk of psoriasis development [13]. In this study, CCB and thiazide use was associated with higher psoriasis incidence after adjusting for the abovementioned covariates, including hypertension status. Thus, use of CCB or thiazides might be a significant predictor of the development of psoriasis. In the subgroup analyses, however, among subjects aged < 65 years, not those aged ≥ 65 years, the associations were found between the use of CCB or thiazide and the risk of psoriasis. Therefore, further studies considering age or sex could be needed to confirm the association with antihypertensive medication use and psoriasis incidence.

In this study, subgroup analysis revealed that patients with hypertension aged < 65 years had a higher risk of developing psoriasis. Gelfand et al. showed that young patients with severe psoriasis have increased cardiovascular mortality compared with elderly individuals with severe psoriasis [32]. The increased prevalence of risk factors, including obesity, diabetes mellitus, or dyslipidemia, related to inflammation and oxidative stress increase the risk of hypertension development in younger adults [33], and these are similar to the pathologic conditions that contribute to the development of psoriasis [14], whereas the pathophysiology of hypertension in the elderly includes age-related changes such as elastic tissue degradation and the deposition of calcium in large conduit arteries [34]. Further investigations are warranted to clarify the effects of hypertension management including lifestyle modifications to decrease inflammation and oxidative stress on psoriasis incidence among hypertensive patients aged < 65 years.

The strengths of this study were that the data were collected through the 11-year longitudinal cohort and were based on a large sample size of 42,726 hypertensive patients and 213,630 controls. It is also the first cohort study to investigate the association between hypertension, antihypertensive medication use, and risk of psoriasis incidence in a representative nationwide sample of the South Korean population. However, this study had some limitations. Firstly, the diagnoses of hypertension, psoriasis, and comorbidities such as diabetes and dyslipidemia were identified using ICD-10 codes using claims databases. Therefore, coding errors and/or misclassification errors such as mismatching or misclassifying the target patients due to the nature of claims data could be possible. However, a validation study of the diagnostic codes of the KNHIS claims database revealed that approximately 70% of the diagnosis codes from the KNHIS claims records coincided with those from medical records [35]. Secondly, we were unable to stratify the baseline severity of hypertension for each patient, and information on psoriasis severity was not available in the claims database. Thirdly, we did not assess the dosage information or combination therapy of antihypertensive medication, which might be critical in determining the extent of psoriasis risk. Fourthly, some confounding factors, including weight, body mass index, and behavioral risk factors, such as smoking, alcohol consumption, and physical activity, were not available due to the limited clinical information present in the claims database.

In conclusion, the risk of psoriasis incidence was increased among patients with hypertension. A prior history of hypertension among subjects aged < 65 years, and CCB or thiazide use were associated with an increased risk of psoriasis incidence. These finding provide insights into the association between hypertension and the risk of psoriasis incidence. However, further studies are warranted to clarify whether hypertension is associated with the incidence of psoriasis.

## References

[1] Nestle FO, Kaplan DH, Barker J. Psoriasis. N Engl J Med. 2009; 361:496–509. 10.1056/NEJMra0804595 19641206

[2] Chang YT, Chen TJ, Liu PC, Chen YC, Chen YJ, Huang YL, et al Epidemiological study of psoriasis in the national health insurance database in Taiwan. Acta Derm Venereol. 2009; 89:262–266. 10.2340/00015555-0642 19479122

[3] Kubota K, Kamijima Y, Sato T, Ooba N, Koide D, Iizuka H, et al Epidemiology of psoriasis and palmoplantar pustulosis: a nationwide study using the Japanese national claims database. BMJ Open. 2015; 5:e006450 10.1136/bmjopen-2014-006450 25588781 PMC4298108

[4] Ding X, Wang T, Shen Y, Wang X, Zhou C, Tian S, et al Prevalence of psoriasis in China: a population-based study in six cities. Eur J Dermatol. 2012; 22:663–667. 10.1684/ejd.2012.1802 22910173

[5] Han JH, Lee JH, Han KD, Seo HM, Bang CH, Park YM, et al Epidemiology and Medication Trends in Patients with Psoriasis: A Nationwide Population-based Cohort Study from Korea. Acta Derm Venereol. 2017 10.2340/00015555-2877 29265167

[6] Michalek IM, Loring B, John SM. A systematic review of worldwide epidemiology of psoriasis. J Eur Acad Dermatol Venereol. 2017; 31:205–212. 10.1111/jdv.13854 27573025

[7] Yeung H, Takeshita J, Mehta NN, Kimmel SE, Ogdie A, Margolis DJ, et al Psoriasis severity and the prevalence of major medical comorbidity: a population-based study. JAMA Dermatol. 2013; 149:1173–1179. 10.1001/jamadermatol.2013.5015 23925466 PMC3800487

[8] Takeshita J, Grewal S, Langan SM, Mehta NN, Ogdie A, Van Voorhees AS, et al Psoriasis and comorbid diseases: Epidemiology. J Am Acad Dermatol. 2017; 76:377–390. 10.1016/j.jaad.2016.07.064 28212759 PMC5731650

[9] Kitamura A, Yamagishi K, Imano H, Kiyama M, Cui R, Ohira T, et al Impact of Hypertension and Subclinical Organ Damage on the Incidence of Cardiovascular Disease Among Japanese Residents at the Population and Individual Levels- The Circulatory Risk in Communities Study (CIRCS). Circ J. 2017; 81:1022–1028. 10.1253/circj.CJ-16-1129 28367846

[10] Armstrong AW, Harskamp CT, Armstrong EJ. The association between psoriasis and hypertension: a systematic review and meta-analysis of observational studies. J Hypertens. 2013; 31:433–442; discussion 442–433. 10.1097/HJH.0b013e32835bcce1 23249828

[11] Armstrong AW, Lin SW, Chambers CJ, Sockolov ME, Chin DL. Psoriasis and hypertension severity: results from a case-control study. PLoS One. 2011; 6:e18227 10.1371/journal.pone.0018227 21479272 PMC3066207

[12] Qureshi AA, Choi HK, Setty AR, Curhan GC. Psoriasis and the risk of diabetes and hypertension: a prospective study of US female nurses. Arch Dermatol. 2009; 145:379–382. 10.1001/archdermatol.2009.48 19380659 PMC2849106

[13] Wu S, Han J, Li WQ, Qureshi AA. Hypertension, antihypertensive medication use, and risk of psoriasis. JAMA Dermatol. 2014; 150:957–963. 10.1001/jamadermatol.2013.9957 24990147 PMC4184206

[14] Azfar RS, Gelfand JM. Psoriasis and metabolic disease: epidemiology and pathophysiology. Curr Opin Rheumatol. 2008; 20:416–422. 10.1097/BOR.0b013e3283031c99 18525354 PMC3746494

[15] Cohen AD, Weitzman D, Dreiher J. Psoriasis and hypertension: a case-control study. Acta Derm Venereol. 2010; 90:23–26. 10.2340/00015555-0741 20107721

[16] Armesto S, Coto-Segura P, Osuna CG, Camblor PM, Santos-Juanes J. Psoriasis and hypertension: a case-control study. J Eur Acad Dermatol Venereol. 2012; 26:785–788. 10.1111/j.1468-3083.2011.04108.x 21569114

[17] Phan C, Sigal ML, Lhafa M, Barthelemy H, Maccari F, Esteve E, et al Metabolic comorbidities and hypertension in psoriasis patients in France. Comparisons with French national databases. Ann Dermatol Venereol. 2016; 143:264–274. 10.1016/j.annder.2015.06.024 26969480

[18] Takeshita J, Wang S, Shin DB, Mehta NN, Kimmel SE, Margolis DJ, et al Effect of psoriasis severity on hypertension control: a population-based study in the United Kingdom. JAMA Dermatol. 2015; 151:161–169. 10.1001/jamadermatol.2014.2094 25322196 PMC4728300

[19] Sowers JR. Hypertension, angiotensin II, and oxidative stress. N Engl J Med. 2002; 346:1999–2001. 10.1056/NEJMe020054 12075063

[20] Guzik TJ, Hoch NE, Brown KA, McCann LA, Rahman A, Dikalov S, et al Role of the T cell in the genesis of angiotensin II induced hypertension and vascular dysfunction. J Exp Med. 2007; 204:2449–2460. 10.1084/jem.20070657 17875676 PMC2118469

[21] Sriramula S, Haque M, Majid DS, Francis J. Involvement of tumor necrosis factor-alpha in angiotensin II-mediated effects on salt appetite, hypertension, and cardiac hypertrophy. Hypertension. 2008; 51:1345–1351. 10.1161/HYPERTENSIONAHA.107.102152 18391105 PMC2736909

[22] Madhur MS, Lob HE, McCann LA, Iwakura Y, Blinder Y, Guzik TJ, et al Interleukin 17 promotes angiotensin II-induced hypertension and vascular dysfunction. Hypertension. 2010; 55:500–507. 10.1161/HYPERTENSIONAHA.109.145094 20038749 PMC2819301

[23] Karbach S, Croxford AL, Oelze M, Schuler R, Minwegen D, Wegner J, et al Interleukin 17 drives vascular inflammation, endothelial dysfunction, and arterial hypertension in psoriasis-like skin disease. Arterioscler Thromb Vasc Biol. 2014; 34:2658–2668. 10.1161/ATVBAHA.114.304108 25341795

[24] Cheng H, Li Y, Zuo XB, Tang HY, Tang XF, Gao JP, et al Identification of a missense variant in LNPEP that confers psoriasis risk. J Invest Dermatol. 2014; 134:359–365. 10.1038/jid.2013.317 23897274

[25] Nakada TA, Russell JA, Wellman H, Boyd JH, Nakada E, Thain KR, et al Leucyl/cystinyl aminopeptidase gene variants in septic shock. Chest. 2011; 139:1042–1049. 10.1378/chest.10-2517 21330387

[26] Esteban V, Ruperez M, Sanchez-Lopez E, Rodriguez-Vita J, Lorenzo O, Demaegdt H, et al Angiotensin IV activates the nuclear transcription factor-kappaB and related proinflammatory genes in vascular smooth muscle cells. Circ Res. 2005; 96:965–973. 10.1161/01.RES.0000166326.91395.74 15831814

[27] Elder JT. Genome-wide association scan yields new insights into the immunopathogenesis of psoriasis. Genes Immun. 2009; 10:201–209. 10.1038/gene.2009.11 19262574 PMC2683580

[28] Ena P, Madeddu P, Glorioso N, Cerimele D, Rappelli A. High prevalence of cardiovascular diseases and enhanced activity of the renin-angiotensin system in psoriatic patients. Acta Cardiol. 1985; 40:199–205. 3887825

[29] Morris BJ. Renin, genes, microRNAs, and renal mechanisms involved in hypertension. Hypertension. 2015; 65:956–962. 10.1161/hypertensionaha.114.04366 25601934

[30] Cohen AD, Bonneh DY, Reuveni H, Vardy DA, Naggan L, Halevy S. Drug exposure and psoriasis vulgaris: case-control and case-crossover studies. Acta Derm Venereol. 2005; 85:299–303. 10.1080/00015550510032823 16191849

[31] Cohen AD, Kagen M, Friger M, Halevy S. Calcium channel blockers intake and psoriasis: a case-control study. Acta Derm Venereol. 2001; 81:347–349. 11800142 10.1080/000155501317140061

[32] Gelfand JM, Neimann AL, Shin DB, Wang X, Margolis DJ, Troxel AB. Risk of myocardial infarction in patients with psoriasis. Jama. 2006; 296:1735–1741. 10.1001/jama.296.14.1735 17032986

[33] De Venecia T, Lu M, Figueredo VM. Hypertension in young adults. Postgrad Med. 2016; 128:201–207. 10.1080/00325481.2016.1147927 26821528

[34] Franklin S. Elderly hypertensives: how are they different? The journal of clinical hypertension. 2012; 14:779–786. 10.1111/j.1751-7176.2012.00703.x 23126350 PMC8108853

[35] Song SO, Jung CH, Song YD, Park CY, Kwon HS, Cha BS, et al Background and data configuration process of a nationwide population-based study using the korean national health insurance system. Diabetes Metab J. 2014; 38:395–403. 10.4093/dmj.2014.38.5.395 25349827 PMC4209354

